# International insights into peer support in a neonatal context: A mixed-methods study

**DOI:** 10.1371/journal.pone.0219743

**Published:** 2019-07-31

**Authors:** Gill Thomson, Marie-Clare Balaam

**Affiliations:** 1 Maternal and Infant Nutrition & Nurture (MAINN), School of Community Health and Midwifery, University of Central Lancashire, Preston, Lancashire, United Kingdom; 2 School of Education, Health and Social Studies, Dalarna University, Falun, Sweden; 3 ReaCH, School of Community Health and Midwifery, University of Central Lancashire, Preston, Lancashire, United Kingdom; Newcastle University, UNITED KINGDOM

## Abstract

Peer support is a widely used intervention that offers information and emotional support to parents during their infant’s admission to the neonatal unit and/or post-discharge. Despite its widespread use, there are no comprehensive insights into the nature and types of neonatal-related peer support, or the training and support offered to peer supporters. We aimed to bridge these knowledge gaps via an international study into neonatal peer support provision. A mixed-methods study comprising an online survey was issued to peer support services/organisations, and follow-up interviews held with a purposive sample of survey respondents. Survey/interview questions explored the funding, types of peer support and the recruitment, training and support for peer supporters. Descriptive and thematic analysis was undertaken. Thirty-one managers/coordinators/trainers and 77 peer supporters completed the survey from 48 peer support organisations/services in 16 different countries; with 26 interviews undertaken with 27 survey respondents. We integrated survey and interview findings into five themes: ‘background and infrastructure of peer support services', ‘timing, location and nature of peer support’, ‘recruitment and suitability of peer supporters’, ‘training provision’ and ‘professional and emotional support’. Findings highlight variations in the types of peer support provided, training and development opportunities, supervisory and mentoring arrangements and the methods of recruitment and support for peer supporters; with these differences largely related to the size, funding, multidisciplinary involvement, and level of integration of peer support within healthcare pathways and contexts. Despite challenges, promising strategies were reported across the different services to inform macro (e.g. to facilitate management and leadership support), meso (e.g. to help embed peer support in practice) and micro (e.g. to improve training, supervision and support of peer supporters) recommendations to underpin the operationalisation and delivery of PS provision.

## Introduction

Globally approximately 1 in 10 babies (~15 million) are born premature (<37 weeks gestational age) [1]. Premature infants as well as those who are born term (>37 weeks gestational age) but who are sick often require admission to a neonatal unit for appropriate medical support. While the numbers of term infants who require hospitalisation are not generally reported, there are disparities in the rates of premature birth across different countries, with the highest rates of prematurity occurring in South Asia and sub-Saharan Africa [2]. However, despite these variations, the problem of prematurity is not confined to low income countries, with USA and Brazil being in the top 10 countries with the highest rates of premature births [2].

The hospitalisation of a premature and/or sick infant can be a particularly distressing experience for parents. Parents can experience early and repeated separation from their infants, which together with concerns for the infant’s wellbeing, can lead to high levels of guilt and helplessness [3–5]. Between 30–76% of parents experience stress and/or depressive symptoms during their infant’s admission in the neonatal unit [6–8] and high rates of post-traumatic stress disorder in parents have been reported [9–11]. A recent study undertaken by BLISS with 589 parents reported that 35% of respondents considered their mental health to be ‘significantly worse’ following the neonatal experience, and 23% had been diagnosed with anxiety [12]. While the need for psychological support for parents is well reported [13,14], available evidence indicates that formal provision is insufficient and often lacking [15,16]. An intervention that is commonly used in neonatal care to promote parental wellbeing is peer support (PS). PS differs from support provided within personal or professional networks as it brings together non-professionals (i.e. peers) and individuals who have had similar experiences (i.e. of having a premature/sick infant) and often share the same sociodemographic characteristics to provide mutual support [17]. PS involves informational, emotional, practical or social support [17] which can be provided in home, hospital and/or community locations, and delivered in groups, pairs, face to face, via the telephone, Short Message Service or via social media (i.e. Facebook) [18,19]. While Cochrane reviews of PS provision in parenting-related contexts [20, 21] have highlighted a lack of high quality evidence, the results indicate a positive influence of peer support on psychological (i.e. reduced depression [20]) and health (i.e. increased breastfeeding [21]) outcomes. The potential salutary effects of PS for recipients are believed to be created through reduced isolation, normalising affects, reducing the impact of stressors and positive role modelling [17].

Research into the impact of PS provision in a neonatal context report improved parental wellbeing though increased confidence [22,23] and self-esteem [24] and decreased stress, anxiety and depression [23–25]. A study by Minde et al. [26] found that parents who received support from peers via discussion groups visited their infants more often and displayed more positive parent-infant interactive behaviours. Furthermore, a randomised control trial undertaken by Preyde [27] found that mothers who received PS were more confident in their parenting abilities and more able to understand the medical condition of their infants compared to mothers in the control group. While this research, most of which has been undertaken in the USA, identifies promising insights, it is important to highlight that these studies often relate to planned interventions with heterogeneous designs, rather than organically developed PS services. To date there has been no comprehensive international study to elicit the types of PS provided in a neonatal context [14].

As indicated above, research undertaken with recipients of PS generally report positive findings [28, 29]. However, research findings into the impact of providing PS on the peer support worker are variable. From a positive perspective, research has identified how peer supporters can reap benefits through enhanced knowledge, feelings of personal control, confidence and improved wellbeing [30–32]. However, other studies identify that peer supporters can feel overburdened when operating as a replacement for professional support [33] and can face tensions in the relationships they form with recipients, i.e. the extent to which they operate as a ‘professional’ or ‘friend’ [9, 30]. A meta-synthesis to explore the impact of PS in the context of perinatal mental illness also identified difficulties when there were differences in the peer supporters and recipients experiences [34]. Insights from the wider PS literature suggest that peer supporters can experience physical and emotional stress, resentment and emotional contagion when providing support to those who have a similar background [30, 35–39]. These findings raise important questions in terms of how peer supporters, with a background of adversity and potential to be re-traumatised, are trained and supported to provide this emotion-based role. While the need for training, supervision and support for peer supporters is highlighted in the literature [14], there has been no research undertaken to assess the types of support offered within neonatal PS services, nor how such support should be provided.

In this paper we report insights from an international study with neonatal PS services/organisations. Due to the current paucity of research in this area, the aim was to elicit insights into the scope, nature and types of PS provided as well as the training, support and supervision of peer supporters. A key purpose was to generate a greater understanding of how PS models ‘work’ within a range of different settings and to identify recommendations and transferable lessons to underpin the operationalisation and delivery of PS.

## Methods

### Study design

A mixed-methods study comprising online surveys and interviews was undertaken between July, 2016-March, 2018. Through early discussions with colleagues it became evident that we needed a clear definition of PS. Our focus was on formal, organised PS provision, rather than PS that can naturally occur (e.g. through informal peer contacts) or professional-led provision (e.g. PS groups facilitated by a professional). Following discussions with international academics and professionals who work within a PS and/or neonatal context we developed a definition of PS (see Table 1) which was subsequently used to identify eligible services.

**Table 1:** Peer support definition.

**All** of the criteria in point one **AND any** of the criteria in point two.
**1) Peer supporters (parent supporters/parent counsellors/parent mentors/parent veterans) are parents:**
a) who have had a sick/premature baby that was cared for in a neonatal unit
b) who provide support to parents who are experiencing high risk pregnancies and/or whose infants are currently being cared for on the neonatal unit or have been discharged
c) who provide support to parents (which could include giving information, practical, emotional and/or social types of support)
d) who offer support via face to face, telephone/text or social media
e) who offer one-to-one or group-based support in hospital or community settings
f) who have received 'some' training/guidance to provide support to other parents
g) who may provide support on a voluntary or paid basis
**AND**
**2) The peer support service/programme is organised/coordinated/provided by any of the following:**
a) National/local services or organisations (such as parenting, breastfeeding or voluntary organisations)
b) Hospital staff
c) Other health and social-care professionals

### Data collection

Two online surveys were initially developed in English, using the Bristol Online secure survey platform—one for managers/trainers/coordinators (MTCs) and one for peer supporters—to allow their related but different perspectives to be captured (see S1 Appendix for both survey versions). Survey questions were developed by drawing on existing literature [14] as well as the authors’ expertise in this area. GT (psychology background) and MCB (social scientist) have undertaken research and evaluation-based projects into PS for breastfeeding women, vulnerable population groups (e.g. those experiencing mental health, domestic violence) and women who have experienced birth trauma. GT is also a steering group member of a multidisciplinary network that aims to improve neonatal care and outcomes for parents and infants (SCENE network). Both surveys included questions related to the types of PS support offered, the nature and timing of PS training, supervision and mentoring, emotional support available to peer supporters and facilitators and barriers to effective PS delivery. In the MTC survey we included additional questions on the background, funding and peer supporter criteria and recruitment processes. The surveys were initially piloted with six professionals and academics who work in neonatal and/or PS services with slight alterations made subsequently. Survey questions included pre-defined options and free text boxes. Participants who were willing to take part in a follow-up interview (to be undertaken in English) were asked to provide their contact details.

A range of methods was used to distribute the survey. First, we sent an introductory email to existing UK, European and international contacts in PS organisations, international neonatal and maternity care research networks (i.e. SCENE, EU COST Action: IS1405), the European Foundation for the Care of Newborn Infants (EFCNI) parenting organization, and to neonatal parent related/PS organisations we identified via internet searches. A recruitment advert was issued via social media (i.e. on relevant Facebook groups and Twitter) with participants asked to contact us direct if they were interested in participating. Snowballing methods were also used whereby PS providers were asked to share the information with other services/organisations as appropriate.

During initial communications with a named contact in the PS organisation/service (i.e. the individual who responded to our introductory email or recruitment advert), we checked whether their service met our definition of PS. If the service met our criteria, we provided the contact with participant information in English or if needed, in translated form. Participant information was translated into Spanish, Portuguese, French, Danish and Finnish by colleagues and volunteers, and then checked for accuracy by another native speaker. A participant information sheet and links to both versions of the survey (MCT and peer supporter) in the requested language was sent to our named contact, with a request for the information be distributed to relevant members of their organisation/service.

At the start of the survey, participants were asked to read a series of consent statements to confirm they understood why the study was being undertaken, the voluntary nature of participation, how to withdraw their data from the study, how confidentiality would be maintained, and the use of anonymised data. Once participants had indicated their agreement (by ticking a box), they could then proceed to answer the survey questions.

Follow-up interviews were undertaken with purposively selected individuals. While 43 survey participants (n = 19 MCTs; n = 24 peer supporters) agreed to take part in an interview, we selected individuals who had different roles (e.g. MCTs, peer supporters) and who were involved in different models of PS (e.g. provided by national or local organisations/services, delivered) in different settings. Telephone or online (via Skype) semi-structured interviews were undertaken by GT and MCB. The interviews took between 30–78 minutes to complete and were audio recorded. All interviews were transcribed in full for analysis purposes.

### Data analysis

Descriptive statistics of survey responses were undertaken using SPSS v.24. All data were then analysed thematically with the support of MAXQDA software. Braun and Clark’s [40] thematic approach was used which involved reading and re-reading the data to enable familiarisation. The data was then organised and mapped into codes, which were then merged into themes that represented the body of the data. The process involved re-reading of the data and the emergent themes to ensure accuracy and authenticity, with re-organising and refinement undertaken where necessary. Both authors were involved in all analytical decisions.

### Ethics

Ethical approval for the study was granted by the Science, Medicine, Engineering, Medicine and Health ethics sub-committee from the authors’ institution (STEMH 209).

### Funding

The study was funded through a British Academy Leverhulme Small Grants award to the lead author.

### Findings

Thirty-one MCTs and 77 peer supporters completed the survey from 48 different PS services in 16 different countries. Twenty-seven participants (13 MCTs and 14 peer supporters from 19 different PS organisations/services) took part in 26 interviews—one interview involved an MCT and peer supporters from the same PS service (see Table 2).

**Table 2:** Overview of numbers of participants and types of participation by country of PS service.

Country (n = 16)	Number of PS services (n = 48)	Survey respondents		Interview participants	
		MCTs (n = 31)	Peer supporters (n = 77)	MCTs (13)	Peer supporters (14)
America	8	7	15	3	3
Australia	6	4	3	-	1
Belgium	1	1	-	1	-
Canada	4	2	10	-	2
Denmark	1	-	1	-	-
England	7	9	9	4	3
Estonia	2	1	3	2	1
Global	1	-	1	-	-
Finland	4	2	6	-	-
Lithuania	1	-	1	-	-
New Zealand	3	-	3	-	-
Northern Ireland	1	1	9	1	1
Mexico	1	1	2	-	1
Republic of Ireland	1	1	2	1	-
Rwanda	1	-	1	-	-
Scotland	2	-	3	1	-
Spain	4	2	8	-	1

The thirty-one MCTs held positions such as Chief Executive Officer, Director, President, Service Manager and Trainer/Coordinator. Sixty-seven of the peer supporters were volunteers, and the remainder (n = 10) were employed in a paid capacity.

As information on the background, funding and peer supporter criteria and recruitment processes were collected via MCTs surveys only, these data were available for 26 services/organisations (see S2 Appendix). The remaining information captured across both surveys in relation to types of PS support, training, supervision/mentoring and emotional support provided to peer supporters were amalgamated to report insights for the 48 participating PS services (see S3 Appendix). While variations from respondents in the same organisation were noted, likely due to different roles (e.g. peer supporters based in hospital or community settings), we triangulated responses (from across survey, interviews) to elicit ‘overall’ insights.

The information collected via survey and interview data are reported in five main themes and associated sub-themes (see Table 3). A selection of quotes are included together with a participant identifier that indicates the participant role (MCT or peer supporter), country, project number and data source (survey or interview).

**Table 3:** Overview of themes and sub-themes.

Theme	Sub-themes
Background/infrastructure of peer support services	- Service focus and scope - Professional backgrounds - Funding
Timing, location and nature of peer support	- Timing and types of support - Peer support delivery - Parent-peer matching and relationships - Integration with wider professionals
Recruitment and suitability of peer supporters	- Recruitment methods and criteria - Assessment methods
Training provision	- Availability and length of training - Training content - Training providers
Professional and emotional support	- Supervisory and mentoring provision - Access to emotional support

### Background/Infrastructure of peer support services

#### Service focus and scope

Most PS services were provided by parenting/voluntary organisations and six had been developed in-house by individual hospital trusts. A few (n = 3) focused on breastfeeding/infant feeding support and the remainder offered a ‘listening ear’ type service. Insights from open text survey responses and qualitative interviews reported how the ethos of PS encompassed listening to parents concerns, reducing isolation, empowering parents to be more confident, assertive, and actively involved in their infant’s care, and to direct parents to other available support and services:

Lots of listening, really, is what it is. Asking questions, allowing families to be more empowered in their role as a parent in the hospital right? Helping them understand kind of what their role is, and how they can be involved in their child’s care. Because the more they do that, the safer their child is going to be and a better chance that their infant is going to have a better outcome. So what we do through these visits as well is really try to empower parents to be a better partner in terms of their child’s care in the hospital. (MCT1, Canada_1, Interview)

Most services had been in operation for 5+ years (61.5%) and two for 20+ years; the number of peer supporters employed/providing support ranged from 2 to >1,000 (with difficulties in estimating PS numbers due to changing commitments reported). Approximately 30% of services used a mixture of paid/unpaid peer supporters and the remaining services (69.2%) involved volunteers only. Fifteen (57.7%) services only employed peer supporters who had their own experience of having a premature/sick infant, and the remainder (42.3%) employed a combination of those who did and did not have personal experience. Some organisations considered experiential accounts to be essential; *‘first and foremost we’re looking for someone who’s actually been in hospital and had a hospital experience’* (Peer supporter 45, USA_27, Interview); whereas for others, a more balanced approach of employing those who demonstrated the ‘right’ qualities were reported (discussed further below).

While all included PS services provided support to parents of premature/sick infants, a number of the larger PS services extended their reach to include support to other family members (64.6%) and/or siblings (47.9%). Approximately 46% provided support to health professionals, for example, through training and workshop events. Two of the services also detailed ‘others’ they supported, i.e. individuals who had been premature infants and family friends.

#### Professional backgrounds

We asked the MCTs to record the professional background of their management committee/board of trustees. Six (23.1%) comprised parents/experienced peer supporters only, and all the others had at least one member from a clinical background (e.g. neonatal nurse, neonatologist, general practitioner/family doctor, clinical psychologist, paediatrician, midwife, occupational therapist, physiotherapist). Seven of the larger PS organisations also had representatives from other professional groups on their management board such as social work, chaplaincy or company lawyer. An interdisciplinary management/advisory group was perceived to be important for acquiring information and resources, operationalising PS, and/or advocating change:

You have to have the professionals that are going to support the programme. You have to have people that are valued and important, including the powers that make the budget (PS61, USA_35, Interview)

Many of the organisations/services had also made connections with other PS providers or larger parenting organisations for support and guidance:

We partner with organisations that do things we don’t do. Thankfully to the internet, we can do that. We don’t do a lot of vlogging and internet stuff and YouTube-ing and teaching parents that way, but other organisations do do that in our country, so we partner with them. So we partner with an organisation called (PS group name) and (PS group). (PS23, USA_14, Interview)

However, a few MCTs highlighted ongoing challenges of establishing PS within fragmented health systems, and when there was no unified presence for PS. One participant referred to how engaging support from other services, professionals and parents was essential to lobby for change:

We’re [PS services] fighting the same cause so then we’re thinking, maybe if we join together like that, giving people the liberty to follow on their objectives, at least we’ll be united and then, once we’re united, we will have that—a voice of influence to then have governments starting to do something. […] When you join forces, health professionals and the parent, then you see that the government has got no choice but to do something. (MCT31, Belgium_14, Interview)

#### Funding

Approximately 30% of the services received funding from hospital/public health funds/commissions, 46.2% relied on grants/donations, and 11.5% received funds from both sources (7.7% received no funding and 3.8% did not provide an answer). Many participants highlighted funding as a contentious issue. The larger organisations with more formalised provision (i.e. clearly defined roles, reporting systems and infrastructure) and who had close connections with health services were more able to access funding, such as through commissioning processes and meeting eligibility criteria for large grant applications. Conversely, many of the smaller organisations struggled, i.e. *‘our organisation doesn’t seem to fit into any of the categories to get government grants’* (Peer supporter 13, Australia_6, Interview) and rather relied on fundraising and/or donations. Some highlighted how their service ran on a *‘shoe string’* with minimal costs involved, due, e.g. peer supporters donating their own resources. The number of services/organisations who offered PS in the same geographical area also meant high competition for funding applications. Challenges in re-negotiating funding issues on a regular (i.e. annual, biannual) basis, particularly in a context of hospital/public health austerity were reported.

Some of the larger, more established PS services had developed resources, e.g. DVDs, essential guides, baby-related items, e.g. Angel gowns (custom made gown for final photos and burial services following an infant bereavement), sought sponsorship, and coordinated conferences, to generate income to fund PS. For others, a scarcity of funding had many negative impacts including: reduced opportunities to recruit and train peer supporters, lack of childcare provision for peer supporters (thereby limiting opportunities to provide PS), restricted geographical reach (e.g. due to insufficient funds to pay travel expenses), and limited promotion of the service:

For us being a not for profit, it comes down to being able to afford more training, in an ideal world I would like my staff to do refresher course every year. (Peer supporter 39, Australia_36, Survey)

### Timing, location and nature of peer support

#### Timing and types of support

Overall, 45.8% of the services provided support across the perinatal period, with the majority offering support during the intrapartum (91.7%) and/or postnatal period (79.2%). In 87.5% of the included services, peer supporters were able to support the same parents overtime, with continuity of care enhanced (where possible) through case reports (providing details of the peer-parent contacts) being shared within the wider PS team. Some community-based services had set time-periods for PS, i.e. up to three months or two years, whereas others had fluid boundaires to offer support ‘*for as long as needed‘*. The end of the parent-peer relationship at a fixed time-point, or when parents had decided they no longer needed support could be *‘abrupt‘*, with some participants identifying how a sensitive closure to the relationship was more appropriate:

I'll mention it to my co-ordinator, she will make an appointment to go out and see the family. Because they know that sooner or later it's going to come to an end and everyone is in agreement and then you set a date. You don't make it for the next week, it tends to be for two or three weeks time and so that is just a period of calming down and saying goodbye, rather than “by the way we're finished Thursday, bye-bye”. You know, it's done on a proper basis, as it were. (PS11, Ireland_2, Interview)

Almost 98% of the services offered information to parents via in-house or wider evidence-based resources (e.g. leaflets, web pages) and details of where to access local help and guidance. Forty-five offered emotional support (93.8%) which generally involved active listening, empathising with concerns, reassurance, and helping to normalise parental experiences and responses. While social support, such as social visits or attending appointments with parents was only offered in ~46% of PS services, participants emphasised how they connected parents in their locality, such as through organised in-hospital and community events, groups, and via social media. Approximately 54% of services offered practical based assistance such as help to provide direct care for their infants, basic household tasks, funding, transportation, childcare, essential items (i.e. breast pumps) and pamper packs.

#### Delivery of peer support

While four services provided support via online methods (i.e. Facebook, web pages) only, others utilised a range of mediums such as one-to-one contacts (87.5%), group-based support (70.8%), social media/online (77.1%), written information (i.e. emails, leaflets) (62.5%) and/or telephone/texts (72.9%). Face-to-face support was generally provided in hospital (77.1%) or community (56.3%) locations; only ~27% of services offered home visits, often due to insurance costs. Participants considered that varied contact methods promoted accessibility and could enable intentional and unintentional opportunities for parents to seek and receive support:

Immediately when they contact us by the shop [online shop selling specialised items for premature babies] I establish a relationship with them [via Facebook]. We discovered that was very good way to get in touch with many people. (MCT25, Mexico_30, Interview)

In-hospital support was considered essential due to the demanding and difficult technological neonatal environment and high levels of parental stress and anxiety. It also provided a proactive means to inform parents about other support options post-discharge. The transition from a supportive neonatal unit environment, to an isolated home situation was recognised as particularly challenging:

We know that parents post discharge is the biggest period of stress for parents where they feel isolated and where problems begin to emerge. (MCT2, Ireland_2_Interview).

Despite this need, many participants expressed difficulties in providing in-hospital peer support. These difficulties related to the qualities of the peer (e.g. confidence in approaching parents), parental mental wellbeing (i.e. not the *‘right time’* to receive support), space on the neonatal unit and cooperation of health professionals (discussed in more detail below). Also, while not frequently reported, there could be challenges in providing targeted one-to-one community support due to parents not responding to peer contacts. Mixed views of the success (i.e. attendance) of hospital and community group-based support were also reported. Some described vibrant, well-attended hospital and/or community groups, whereas others experienced difficulties associated with costs (i.e. room hire, refreshments), ongoing promotion, reliance on ‘dedicated’ individuals and non-attendance:

You can’t force people to come to groups if they don’t want to come to groups, but that’s fine. They must be either, not in need for support or the support is coming in different ways or some reason why our support is not working. (Peer supporter 15, England_7, Interview)

A key challenge raised by many participants concerned the nature of voluntary work. Peer commitment to provide agreed hours was difficult to enforce, and a source of frustration for service managers, peers, parents and health professionals:

One of the most complained about things from the midwives and also parents at hospital is why is it on one week there might be a peer supporter coming every other day and then the next week there is nobody. Why is it that the women last week got the gold top service where they were peer supported from every angle, and this week it's not available. (Peer supporter 24, England_16, Interview)

Participants highlighted instances of volunteers *‘dropping out‘* of the service, or coordinators *‘wasting time‘* in attempts to monitor peer activity. Others referred to difficulties in organising PS due to poor communication with coordinators/managers; *‘because X [name or organisations/service] is run by volunteers […] you won't an email for several weeks’* (Peer supporter 24, England_16, Interview). These issues were less apparent within the larger PS organisations due to their capacity to recruit and train larger numbers of volunteers. Those who had employed a paid coordinator to form positive relationships and maintain regular communication with peer supporters also reported how this had led to decreased attrition rates.

#### Parent-peer matching and relationships

While peer-parent matching was less easy to achieve within smaller PS services, just over 73% of the PS services either ‘always’ or ‘sometimes’ matched parents to peers. The most common form of ‘matching’ related to the parent/peer having an infant with similar health issues (88.9%). While this suggests that similar experiences rather than shared socio-demographics were super-valued–the primacy of personality factors in facilitating a parent-peer connection was highlighted:

Yes, and then even though they think they've made the right choice by matching me up with a family, they're still got the opportunity to say no, no it's not working. It's not set in stone, if you find out after that initial visit that it's not going to work you say goodbye and you find someone else. (Peer supporter 11, Ireland_2_Interview)

Some peer supporters expressed difficulties in engaging with parents, due to language and cultural differences or different experiences of prematurity, e.g. *‘I do have some difficulties when it comes to the very sick infants who don't have a good prognosis‘* (Peer supporter 77, USA_48, Survey). Whereas other peers considered that general commonalities of their shared experience meant they could connect with parents on some level: *‘there is going to be something with every family that we are going to connect with’* (Peer supporter 69, USA_43_Interview).

There were also differences across the PS services in the expected boundaries of the peer-parent relationship. Some services encouraged personal, friend-type relationships, i.e. sharing phone numbers and open invitations to contact when needed; ‘[peer supporterss] *have to be allowed to have a personal relationship with them* [parents]’ (Peer supporter 23, USA_14, Interview). For others a more professional-based relationship was instilled, whereby peers were advised to not share their personal contact information, and parents needed to seek out support on a reactive basis (unless already agreed in advance). In these occasions, a ‘professional’ distance was believed to be needed to prevent over-involvement and peers feeling overwhelmed:

You’re there as a volunteer. You’re not a friend. You’re a friend to them but you’re not their friend if you know what I mean. There’s a level of professionalism in it. (MCT2, Ireland_2, Interview)

There were also infrequent accounts of parents misusing (i.e. babysitting, paid childcare) or abusing the parent-peer relationship, e.g. *‘peer supporters like servants (seriously*!*) or punching bags*‘ (Peer supporter 69, Canada_43, Survey); thereby *‘blurring the boundaries’* of PS provision.

#### Integration with wider professionals

Overall, integration of PS within a clinical environment was a contentious issue. Many participants referred to how there was/or had been a lack of trust in PS by healthcare providers; with suspicion levied against the types of support that non-qualified individuals provided. Distrust and doubt could lead to professionals ‘gate-keeping‘ which parents the peers could support, not promoting or directing parents to the PS service, not distributing PS resources or peer supporters not being able to access the neonatal unit:

Hospital staff can be suspicious of peer supporters and worried about unqualified people giving case specific advice, or making disputes between staff and parents worse. This needs to be managed carefully or they can undermine the group by not signposting parents towards it. (Peer supporter 15, England_7, Survey)

Misunderstandings of the peer supporer role could lead to peers being expected to perform tasks outside of their role boundaries; ‘*As they* [peer supporter] *got more skilled*, *people were expecting them to do things outside their scope of practice’* (MCT12, England_15, Interview). A further issue related to how much information healthcare professionals could or would share with peer supporters prior to them initating contact with parents. A lack of background information (i.e. infant status, diagnosis, parental mental wellbeing) could lead to peers feeling unprepared, mentally and emotionally; *‘Very unprepared at times when I walk in with absolutely no information as to what’s going on’* (MCT1, Canada_1, Interview).

From a counter perspective, some PS organisations (particularly those who had a multidisciplinary management board) described positive working relationships with hospitals/clinical staff. Participants referred to how these relationships had taken considerable time to come to fruition; e.g. ‘*years of working together‘* and were built incrementally through ‘*building individual networks‘* and personal relationships with different clinical/managerial staff members. These partnerships were built on trust, clear/defined boundaries, an understanding of the peer supporter role, and regular and open lines of communication between the PS service and clinical staff. One service which had developed these relationships reported:

We became successful after that because those doctors and nurses advocated for us to go to other hospitals and do the same thing because in the neonatology world, being so physically close, the neonatologists all know each other—the doctors, they go to conferences together so they talk about us. So they were able to cheerlead, “This group is for real, they know what they’re doing, they’re good at it, let them go”. (Peer supporter 23, USA_14, Interview)

### Recruitment and suitability of peer supporters

#### Recruitment methods and criteria

While the services used various methods to recruit peer supporters, self-recruitment was the most common (46.2%). This included parents who had been in direct receipt of PS, or who learnt about their organisation/service via personal networks or local events joining the PS service.

Just over 53% of the services enforced a minimum period between the peer supporter’s own experience of having a premature/sick baby and providing support to parents. The expected time interval varied between 6 months-3 years across the services, and the majority of peer supporters (86.2%) surveyed had joined the PS service 12 months+ after their own experience of neonatal care. The ‘agreed’ time-frame within the PS services was often based on professional-led recommendations, ‘*making mistakes’* by recruiting volunteers too soon in their emotional journey or had evolved naturally through allowing peers to make their own decisions. Irrespective of the length of time imposed, there was an implicit recognition that healing takes time, and that a peer’s capacity to offer support in the early post-natal period, when dealing with demands of a new infant, and potentially one with compromised/poor health, would be restricted:

We really want our mentors in a place where there is some consistency for the parent that they are mentoring and we’re not saying that you’ve got to be perfect because nobody is but just getting them to a place that we think is more stable, I guess, in terms of their own personal life and the life of their children (MCT8, USA_10, Interview)

Some of the ‘expected’ peer qualities were to be ‘natural supporters’, possess good communication and listening skills, demonstrate compassion, *‘who want to be there for other people’*, and to commit to specified hours of support (e.g. 2, 4 hours, every week, fortnight, etc.). However, overall ~73% of the services had experienced situations when a peer supporter was not suitable due to not being ‘*emotionally ready’*, failing to meet expected peer commitments, professionals not wishing to work with *‘difficult parents’* (based on their prior history), and peer supporters operating outside of PS boundaries (i.e. providing non-evidence based information). In these occasions peers could be counselled out of the service or directed to other areas of activity (e.g. fund raising, online support).

#### Assessment methods

A number of assessment methods were used to assess peer suitability; with these methods designed to elicit the level of emotion displayed by peer supporters when challenged with certain scenarios, how comfortable they were in approaching and communicating with parents, their ability to moderate their stories–to *‘hear more than they talk’*—and capacity to direct and refer, rather than offer ‘advice’ to others. An overview of the methods used across the PS services are detailed as follows:

Interviews: In all bar two of the PS services, peer supporters were interviewed by service/organisation staff and/or multidisciplinary professionals; with the value of ‘specialists’ (i.e. social workers, psychologists) to probe for and identify the peers *‘emotional baggage’* highlighted. Interviews generally followed a similar format of introductions to the service(s), nature of the role, and exploration of individual expectations and commitments to provide PS. Examples of questions used to probe and explore the peer’s ‘readiness’ for PS are detailed in Table 4.

**Table 4:** Example questions used during interviews with peer supporters.

Example questions used during interviews with peer supporters
How do you see yourself as a peer supporter?
How did you get on with the staff?
How did you respond to problems/major problems in the neonatal unit?
What kind of parents will you be willing to support?
How would you be if the first parent you meet in the unit tells you their story with their baby and it's almost exactly the same as yours?
How do you manage your stress and anxiety?

Observations: Observations of peer responses in simulated scenarios (73.1%), i.e. role-playing were a well-utilised and valued technique during the training programme:

We spend a lot of time on active listening, talking about empathy, talking about some of the skills of active listening such as paraphrasing, listening with purpose that kind of thing. Looking at body language, role playing, that kind of thing. (MCT1, Canada_1, Interview)

Almost 70% of services used ‘shadowing’ to assess peer readiness–whereby the peer supporter observed more experienced peers/was observed providing direct support to parents. Ten (55.5%) services provided shadowing for a fixed period, i.e. once or twice, 3 months, etc. Some services had the flexibility to extend the shadowing period as needed, whereas others were unable to do so due to staff reticence and resources. Almost 45% of PS services offered shadowing on a purely flexible basis—up until the peer/observer was sufficiently confident; *‘Until they feel confident enough to do it on their own and their mentor believes they are ready*‘ (MCT6, Australia_7, Survey).

A small number of services employed peers in other activities prior to them offering one-to-one support to parents (e.g. at parent groups, fund raising, compiling and distributing resources). This was reported to be a useful strategy to assess the peer’s potential and motivations:

We give them the option when they come in of looking at all the activities within the organisation and explaining that while the peer support is an option it's an option down the road and trying to find somewhere in the organisation for them to fit when they're doing that sort of bedding in period to give us the opportunity to understand what their strengths are or what their weaknesses are to try and weed out those families who may be in it for the wrong reasons unbeknown to themselves, trying to heal themselves and possibly end up doing more damage. (MCT10, Ireland_12, Interview)

Seeking feedback: All services utilised some form of feedback to assess the peer supporters’ suitability whether from individual peers (53.8%), other peer supporters (65.4%), wider staff members (65.4%) and/or parents (65.4%). Some services that provided targeted support (i.e. one-to-one) maintained close contact with parents and the peer supporter during the early period to assess whether it was a suitable match–with the frequency decreasing once a suitable parent-peer connection had been established. A few of the larger, more established services also requested references (which in one service was from the peer’s paediatrician, *‘to get a sense of how the child has been developing’* and the peer’s adjustment).

Sharing stories: Approximately 94% of services enabled/encouraged peers to share personal accounts during the initial training programme (75.6%), supervision sessions (64.4%), social occasions with peer supporters (37.8%) and counselling sessions (71.1%). Participants considered opportunities for peers to share and reflect on their own experiences to be crucial:

[Training] It’s actually about trying to put people in their shoes but also about a lot to do with self-awareness and self-protection because volunteering does take quite a lot of emotion out of people and if you have been through an emotional journey yourself and you’re the volunteer offering that emotional support, you’ve got to really understand where you sit with that. So really, through encouraging peers to share their experiences, our training tries to understand is where you’re at in your emotional journey. (MCT8, England_8, Interview)

Sharing stories were considered beneficial for assessing peer suitability, facilitating healing, i.e. to enable them to *‘be better supporters of others*’, for peers to understand that ‘*others have different experiences’* and peer bonding. Opportunities to discuss personal accounts provided information to help parent-peer matching (where appropriate) and used to assess if/when peer supporters needed respite, e.g. at anniversaries of their traumatic/distressing account, i.e. infant birthday.

### Training provision

#### Availability and length

Just over 80% (n = 39) of services provided a formal training programme that was usually provided on a face-to-face basis and had been developed in-house via input from other PS services, parents and professionals. The length of initial training offered across the PS services varied (i.e. 30mins– 80hrs), and 20 of the services who provided specific information offered 10+ hours basic training. The larger PS organisations tended to offer longer training sessions and the length of training determined whether it was offered on a single, or over multiple days; with training delivery over different days reported to be an important means to assess peer performance overtime. Overall, sixty-nine (89.6%) peer supporters considered that they had either partly/fully received sufficient training and the remainder responded ‘not at all’ (n = 10, 10.4%). The MCTs expressed similar attitudes with 83.9% (n = 26) agreeing and 16.1% (n = 5) disagreeing that suitable levels of PS training were provided.

In ~58% of services peer supporters had to undergo security checks and additional training such as privacy/confidentiality and/or hospital induction training (e.g. infection prevention, record keeping). Some hospitals also insisted that blood tests and vaccinations were undertaken prior to the peer supporter’s access to the clinical environment. A few participants (notably UK based) complained about inflexible hospital systems/procedures and the lengthy wait for all checks/training to be undertaken:

So it was about September 2015 that I started thinking about it and scoping it out. And then it took months and months getting the application done with X [name of organisation] and then waiting for training, and then you go to the hospital and you have your DBS [Disclosure and Barring Service–criminal records checks] checks and the interviews for them stuff like that. So yes, it wasn’t until May 2016 that I first started actually supporting parents on the unit so it was a long process. (Peer supporter 17, England_7, Interview)

#### Training content

Information on training content was available for 37/39 services. All training programmes provided instruction on the peer supporter role/boundaries of support provision such as to ‘listen’ and direct parents to other forms of support (e.g. clinical, financial, psychological); to make evidence-based rather than personal recommendations and to avoid colluding with parents, e.g. over negative parent-professional interactions:

They gave you some examples of what you might like to do for example pick a doctor that you like and talk about them or if or if you disliked a nurse to date, rather than saying “I don’t want her to ever look after my baby, she’s incompetent”, may be say “well, which nurse do you like?” or maybe go and talk that nurse. Reassure the parents about the professionalism of the staff. (Peer supporter 15, England_7, Interview)

Most training provision included insights into the ‘expected and normal responses of parents who have premature/sick infants’ (86.5%), ‘how to show empathy and understanding’ (78.4%), ‘knowledge of other services/support’ (75.7%) and ‘basic communication and listening skills’ (72.9%). More specialist training, i.e. identifying parents at risk of mental health issues (64.9%), understanding the natural stages of grief/mourning and loss (59.5%) and practical skills training (37.8%) was less common: with competency-based practical instruction more likely offered to peer supporters who provided breastfeeding/infant feeding support. Training programmes included case studies, videos and/or role-play to present real-life situations and scenarios, with qualitative feedback indicating that these were invaluable to learn, reflect and address concerns in a non-judgemental environment:

We spend a lot of time on active listening, talking about empathy, talking about some of the skills of active listening–such as paraphrasing, listening with purpose that kind of thing. Looking at body language, role playing, that kind of thing (MC1, Canada_1, Interview)

#### Training provider

Whilst training was often provided by service/organisation members (91.9%), five involved parents in training delivery. The PS organisations who had established practices within healthcare settings and/or multidisciplinary professionals involved in PS service delivery also employed specialist staff (e.g. social workers, clinicians, counsellors) to deliver/co-deliver the sessions. Some participants expressed the value of specialist input and experiential insights to promote the realities of PS:

But the really useful thing for me was to talk to other volunteers and particularly people who have been volunteering who came to help. So you can say “well have you had a situation when?” or “what would you do if?” (Peer supporter15, England_7, Interview)

Funding issues and lack of infrastructure meant that some of the smaller PS organisations could only offer introductory training (37.5%). The remaining (62.5%) services provided ongoing in-house training, offered e.g. on a monthly or quarterly basis, either embedded within supervisory sessions, or as stand-alone events. Annual updates or ‘ad hoc’ sessions could also be provided, e.g. *‘we circulate webinars*, *videos and provide external training opportunities where appropriate’* (MCT11, England_9, Survey) which while useful for learning opportunities, attendance was not always mandated.

Recurrent areas of training needs reported by the peer supporters concerned interpersonal and communication skills and counselling/crisis skills development. A few also requested further knowledge of neonatal unit processes and procedures and post-discharge support.

### Professional and emotional support

#### Supervisory and mentoring provision

Approximately 69% (n = 33) of the PS services provided some/all peers with formal supervision sessions. Fifty-seven (74.0%) peer supporters reported that they received regular formal supervision, and while ~91% (n = 52) found these sessions to be useful/very useful, ~23% (n = 13) felt further supervision was needed. A few services were either unable to provide supervision or it was only offered at key time-periods (e.g. quarterly) due to a lack of funding. Sixty percent of peers (n = 12/20) who did not receive supervision, wished that this service was available; ‘*for me it’s less about supervision*, *it’s about support’* (Peer supporter 69, Canada_43, Survey).

Supervision could be provided by an experienced peer supporter/organisation member (n = 20/33, 60.6%) and depending on the degree of integration within health service settings, it could also be provided by health or social care professionals (e.g. neonatal staff, social workers, psychologists/counsellors). Timing of supervision varied with some offering formalised meetings (e.g. monthly, quarterly) and an *‘as needed’* approach; with flexible, timely access to supervisors reported to instil confidence, and feelings of safety in peer supporters. Supervision could be provided on a one-to-one and/or group basis via face-to-face, telephone and/or Skype/online dependent on geographical distance, availability of suitable supervisors, funding, how supervision was provided and personal preferences. Group-based supervision offered wider benefits through stimulating discussions, sharing learning and peer-to-peer support. However, as group support could inhibit in-depth discussions; ‘*The lack of material time of the rest of the volunteers prevents us from going deeper into these issues’* (Peer supporter 42, Spain_24, Survey) one-to-one sessions for detailed disclosures and reflection was also considered necessary.

Participants considered supervision important to reinforce skills and learning taught during training, to discuss difficult or challenging experiences (personally or vicariously), assess training needs, and receive feedback and appreciation: ‘[my supervisor is] *super positive and encouraging—always valuing the contribution we make‘* (Peer supporter 57, Canada_21, Survey).

A mentor–a named individual the peer could contact on a daily basis—was provided in the majority of services (n = 41, 85.4%) surveyed; peer supporters that had no mentoring arrangements in place often wished this support was available:

Lots of people are informal mentors but I don't have a formal mentorship and I wish I did. A nurse or social worker would be ideal. (Peer supporter69, Canada_43, Survey)

In 10 (35.4%) services the mentor was an experienced peer supporter and in the remainder (n = 31, 64.6%), mentors included clinicians, counsellors/psychologists and/or social workers. A clinical mentor was considered useful, e.g. to address specific queries. However, busy workloads, staff changes, the extent to which PS was integrated within the care pathways, and lack of training in PS meant that the support was not always available or appropriate. Some participants expressed the need for a clinical *and* PS mentor to address their specific concerns.

#### Access to emotional support

Participants highlighted the *‘exhausting‘*, *‘draing‘*, emotionally-taxing nature of PS, and how *‘release valves‘* to enable peers to debrief and offload were essential. The most common forms of emotional support provided to peer supporters were to contact their supervisor immediately (84.7%) or to talk to other peer supporters (71.1%). More specialist support, i.e. counselling from a trained therapist/psychologist from within or external to the organisation/service was only available in 43.4% and 26% of services respectively. One MCT reported that they would pay for ‘expert’ help for the peer supporter if needed, however, in small, low resourced services, the costs of providing more specialist support was deemed to be *‘prohibitive‘* and *‘unrealistic‘*.

While 85.7% (n = 66/77) of peer supporters, and 83.9% (n = 26/31) of MCTs felt there was sufficient emotional-based support provided, a more mixed response was evident in the qualitative accounts. A lack of regular debriefing could mean peers relying on social networks to provide emotional support; *‘husband always waits up at home for me so if I've had a difficult visit I can 'let it all out*'‘ (Peer supporter 17, England_7, Interview) and a number of participants highlighted this as an area where timely, formal and appropriate provision was required.

Fifty-five (71.4%) peer supporters reported that formal social peer-to-peer events were provided by their organisation/service. These were local and/or state/regional events designed to promote team bonding and to reward peers through pamper gifts, pizza nights, etc. Feedback into the impact of the PS service (such as through sharing parent evaluations) was also commonly provided to ‘*connect them* [peer supporters] *back’* into the service. As the peer supporter role was often fuelled solely by altrustic intent, feedback and appreciation were considered important to sustain their intrinsic motivation.

## Discussion

This study offers a unique international perspective into the background and nature of PS in a neonatal context. While the need for PS among parents of premature/sick infants is highlighted [41–43] insights from the included 48 PS services show wide heterogeneity. There were variations in the types of PS provided, training and development opportunities, supervisory and mentoring arrangements and the methods of recruitment and support for peer supporters; with these differences largely related to the size, funding, multidisciplinary involvement, and level of integration of PS within healthcare pathways and contexts. The heterogeneity of PS provision is reflected in findings from recent UK surveys of PS in other settings, i.e. breastfeeding [43] and support for women with complex psychosocial needs [44]. While on one hand these variations may reflect flexibility and innovation in PS delivery [44], they also create difficulties in terms of replication, and in identifying the ‘core’ essential components required for effective PS provision [45,46].

Funding is a contentious issue for neonatal, as well as other areas of PS delivery [43,44]. However, the positive move demonstrated by some PS services to join forces and share resources with other services is a useful consideration. Our study highlights that successful and effective PS provision requires multiagency collaboration and commitment by a team of relevant and committed professionals [14, 33]; with the expressed difficulties in peer-professional integration concurring with insights from wider PS provision [20,34,45,47]. Some strategies identified via our study, and the work of others, to improve integration include joint training, regular communication to improve relationships, co-working practices, institutional champions and paid parent support coordinators [33, 48]. Moreover, while issues of attrition and sustainability are commonly reported within voluntary based services [33], social opportunities to connect, receive feedback and reenergise peer supporter involvement was identified as an important strategy.

Evidence from across the included services indicates that PS tends to involve information and emotional based support to parents; with the majority providing support in the intrapartum and/or postnatal period. Our findings support wider research that multiple options for PS are needed to meet parents’ needs [14,42,49] and to promote accessible, flexible support. Most of the PS services offered continuity of support by the same peer supporter, thereby reflecting wider literature that emphasises the value of consistent caregivers for meaningful and trust-based relationships to be formed [50–52] and to impact on health-related behaviours [21]. There were differences in the length of support and service level expectations (and associated peer supporter challenges) in the nature of the parent-peer relationship (i.e. professional or friend); with the need to reinforce clear expectations on the role boundaries of peer supporters being reported in wider PS literature [33,36,38,53]. While there were differences in the criteria used to match peer supporters with parents, the most commonly used was similarities in infant’s health status, as recommended by Hall et al. [48]. However, our findings echo those of a recent realist review of one-to-one breastfeeding PS trials [45]. This review identified how shared socio-demographics and backgrounds may be less important than the qualities of the peer supporter [45]. MacLellan et al. [46] also highlight that while peer matching is commonly utilised, the impact of such has not been systematically assessed.

Overall, there were variations in relation to the length of time between the peer supporter’s own experience and providing support to parents. As over two-thirds of the PS services had experienced difficulties in peer supporters being unsuitable, often due to the peer’s unresolved negative emotions, this suggests that a minimum period of at least 12 months should be enforced [14, 54, 55]. Our findings also emphasise the value and need for multiple forms of assessment to assess the peer’s emotional readiness, such as through a multiagency interview panel and careful framing of questions, training delivery staggered over different days, flexible shadowing opportunities and ongoing feedback from recipients, health providers and peer supporters.

Multiple and ongoing opportunities for peer supporters to share their experiences was highlighted as crucial to help resolve adverse responses and prepare peers to provide support for others. The metasynthesis undertaken by MacLellan et al. [46] into the impact of PS on peer supporters reported how sharing experiences with clients could help *‘to give meaning to their suffering’* (p.7). In our study, however, the focus centred on sharing narratives with peers, and moderating their experiences during parent interactions. This difference is likely to be related to the context of care provision, where, for example, a peer supporter sharing insights into their own experience may not be appropriate and may create harm. Ongoing use of role-playing methods may help address these challenges in practice [36]. Furthermore, while all included PS services offered some form of emotional support for the peer supporters, the need for appropriate and flexible support to help resolve any challenges or adverse responses was stressed; a finding reported in other PS literature [14, 55–57].

While our study identified common training content, i.e. role boundaries, communication skills, information on wider support, the amount of training varied significantly, similar to other areas of PS [19,43, 58]. It may be, as recommended by Kemp et al. [59], that practice models together with research findings should be used to develop an accredited training programme. However, our findings also indicate the need for ongoing training, and for more specialist input to help peer supporters engage and support parents who are experiencing high levels of distress. Although concerns about professionalising PS, and extent to which this can deter from the ethos of parent-to-parent support have been highlighted [33,60]. Our findings also indicate a need for regular supervision provided via multiple formats, i.e. one-to-one and group supervision. Mandatory attendance at regular supervision sessions, such as those used within national PS organisations [61] could be introduced; thereby providing opportunities for debriefing and to identify training and emotion-based needs. Furthermore, while a mentor was commonly provided, participants highlighted a need for clinical (for those working within a clinical environment) as well as peer-based support, thereby indicating the value of role-based and context-related ‘expertise’.

There are a number of limitations to our study. First, we were unable to elicit the prevalence of PS provision. We were also unable (despite reminders) to obtain responses from MCTs and peer supporters in the same organisation for all included services. We also identified some variations in responses, e.g. from peer supporters in same organisation, and while we assumed these to be due to their different roles, further clarification may have been useful. While extensive and concerted efforts were made to receive responses from as many PS organisations as possible, it is feasible that our recruitment methods did not have sufficient reach. Follow-up by telephone may also have helped to increase completion rates. Most data were collected from PS services in high income countries, despite active targeting in low/middle income settings (e.g. such as through emails to existing contacts in certain counties, e.g. China, Hungary, India and those associated with the SCENE, COST and EFNCI networks). While representatives from some countries did respond to indicate they were unaware of any PS service that met our definition (i.e. Germany, China, Sweden, Hungary, Bulgaria and Poland) a lack of response from others, despite reminders, meant it was impossible to say whether peer support was less likely to be provided in lower resourced settings. It is also worth mentioning that while included responses from low/middle countries (i.e. Rwanda, Mexico) emphasised a lack of funding and lack of unified presence for PS, these issues were also reported in high-income settings. As we did not use back-translation to verify our translations, this may have affected participant responses, however, all translations were checked by a native speaker, who in most occasions was conversant with the remit/purpose of PS. As we stipulated that all interviews had to be undertaken in English, this may have restricted participation. A further limitation is that this study only focused on the background and infrastructure of PS rather than the impact of these different support models on parent (and infant) outcomes. Further research, such as the use of realist methods, could help to identify how key mechanisms of PS interact with context-related features to influence positive change. The strengths are that this study is the first to elicit insights into a broad range of PS provision from different settings and contexts. The use of follow-up interviews also enabled us to obtain richer, in-depth insights than survey methodologies allow. Through this work we were able to identify macro (e.g. to establish global links/unified presence for PS), meso (e.g. to facilitate and enable effective working practices with healthcare providers and micro (e.g. training and support for peer supporters) level ‘promising’ strategies for other areas to consider (see Table 5). While further research and testing is required, these recommendations offer practical and feasible transferable lessons for neonatal, as well as wider areas of PS delivery.

**Table 5:** Recommendations to inform the organisation and delivery of peer support.

**Management/leadership support:**
To recruit interdisciplinary professionals from all relevant backgrounds where possible onto management committee/board of Directors for the organisation/service;
To enlist the support of key strategic and clinical leads to agree the need for and operationalisation of peer support (procedures, confidentiality agreements, etc.);
Opportunities to co-operate with other peer support providers/organisations may help develop infrastructure, resources (such as training and/or supervision) and funding potential. Connections with national/international associations can also aid service promotion and development;
A lead/coordinator role(s) should be appointed to maintain regular communication with peer supporters and other partners (i.e. healthcare professionals), and to help coordinate/organise service provision;
Stakeholder groups (e.g. members of the clinical team, key professionals, peer support coordinators) should be established at each site, and with regular meetings held;
Some remuneration, i.e. childcare vouchers for peer supporters should be considered to reduce attrition.
**Recruitment and Assessment of Peer Supporters**
Due to the potential for unintended harm and restricted capacity for peer support, there should be a minimum period (e.g. 12 months+) between the peer’s experience of neonatal care and working as a peer supporter;
A formal interview should be held, ideally with members from a range of professional backgrounds (e.g. clinical, peer support, counselling/psychological services) to help make more informed judgements of the candidate’s ‘readiness’ to engage in peer support;
Interview questions should explore the candidate’s own experience of having a sick/premature infant on the neonatal unit as well as their purpose and motivations for applying;
If possible, peers should be employed in other areas of the organisation (e.g. fund raising, charity events) and/or provided with flexible shadowing to support them in the transition from parent to supporter and to assess their suitability, prior to independent support being provided;
A range of feedback methods (e.g. discussions with peer/other peers, professionals and parents) should be used to assess peer suitability, particularly when new to the service.
**Training**
Key training elements should include role/boundaries of peer support, expected and normal responses of parents who have premature/sick infants, how to show empathy and understanding knowledge of other services/support and basic communication and listening skills; Training should be provided over separate days to assess peer suitability overtime;
Ongoing training should be regularly provided (either face-to-face and/or virtual learning communities) to include interpersonal and communication skills, such as through case-discussions, role modelling, and role-play; with online training having added benefits through reduced costs and increased accessibility;
Peer supporters should be involved in identifying their own training needs;
Additional training in mental health awareness and counselling-type skills for crisis resolution and bereavement should be considered;
To consider accreditation of the training programme, thereby offering an additional incentive for peer supporters;
Visits to the neonatal unit could be offered as part of the training programme to: -acclimatize the peer supporters (emotionally and physically) to the unit -raise awareness among staff about the role and purpose of peer support -to aid peers understanding of the neonatal procedures/protocols, etc.
Due to the variability in peer support training, further work to consolidate knowledge and produce key competencies/learning materials would be beneficial;
Training should be provided by interdisciplinary professionals (e.g. social work, counselling/psychology) to offer specific areas of expertise (e.g. communication skills, empathy, coping skills, etc.), and by parents and peers to offer experiential accounts;
Neonatal staff should be involved in the training programme, i.e. to deliver a session on working in the neonatal units and/or ‘meet and greet’ sessions with the peer supporters to raise awareness of the role/purpose of peer support and to forge peer-staff relationships.
**Supervision, Mentoring and Support**
Flexible, and regular access to a supervisor (for emotional and instrumental support) should be provided;
Supervision should ideally be provided on an individual and group basis due to benefits of in-depth disclosures and shared accounts;
Early and regular supervision sessions should be provided when a peer supporter joins the service and/or providing targeted support (i.e. community based support);
Peers should be provided with a key named staff member to contact if they have any concerns or issues, or for debriefing purposes, i.e. following each shift at the unit;
Peers should be assigned a mentor from within the clinical team (where appropriate) who has experience and/or understanding of the peer role in order to: -to facilitate peer’s access to immediate support -to enable debriefing opportunities -to aid team practices and working relationships
Counselling services such as one-to-one sessions should be ideally offered to peer supporters as part of the training, with additional sessions available as required;
Feedback on the peer support service (e.g. from parent, staff evaluations) should be regularly disseminated to peer supporters (e.g. newsletters, supervision meetings) and where possible, regular social opportunities—appreciation events—to increase and/or sustain peer motivation.
Opportunities for peer supporters to share their stories and experiences in a safe, supporting environment is highly valued and should be encouraged where possible/appropriate (e.g. during training, supervision).

## Conclusion

This study provides the first international overview of the ways in which neonatal PS models operate in a range of settings. Using surveys and interviews with those directly involved in organising and providing support, it provides insight into the nature, scope and types of peer support provided in varied geographical and institutional settings. It also addresses an overlooked area concerning the training, support and supervision of peer supporters. While further research is required to identify the key ingredients of effective peer support provision, this work has generated macro, meso and micro level recommendations designed to facilitate the operationalisation of peer support to meet the needs of peer supporters and those they support.

## Supporting information

S1 Appendix MCT and peer supporter surveys. (DOCX)

S2 Appendix Survey responses from MCTs into the background, funding and peer supporter recruitment criteria and processes (n = 26). (DOCX)

S3 Appendix Survey responses from MCTs and peer supporters into the types and timing of peer support and the training, supervision/mentoring and emotional support provided to peer supporters (n = 48). (DOCX)
