# Supplementary material for: International insights into peer support in a neonatal context: A mixed-methods study
Source: PLoS One. 2019 Jul 31;14(7):e0219743. doi: 10.1371/journal.pone.0219743 (PMC6668779; doi:10.1371/journal.pone.0219743)
Supplement: S1 Appendix — (DOCX) [file pone.0219743.s001.docx]

**Questionnaire for managers/coordinators/ trainers of neonatal “peer support” services/programmes**

**Many thanks for your support with this study**

Please could you read the following statements and then click in the box if you have:

Read and understood the information in the information sheet (v.2, 18/7/2016), and have had an opportunity to ask/receive answers to any questions.

Understood that any personal information provided (such as the name of my service/contact information) will not be shared with anyone outside of the research team.

Understood that the findings will be used in reports, journal publications and presentations, but you will not be identified.

Understood that it is up to you if you want to take part or not - you do not have to answer all of the questions - and if you have provided your personal contact details, you will be able to remove your data from the study up until June, 2017.

*Please note that ticking the box indicates your consent/agreement to take part in this study:*

*If you would be willing for us to keep and use your anonymised data for teaching; further research/evaluation; presentations and publications; sharing with other people doing similar studies please click in the box:*

**A. Background Information:**

a.1) What is the name of your “peer support” service/programme? ________________

a.2) What is your role in the “peer support” service/programme (for example, trainer, coordinator, manager)? _______________

a.3) In which country is your “peer support” service/programme? _______________________

a.4) What is your gender? Male/Female

a.5) How long has your “peer support” service/programme been running (please include the month/year when your “peer support” service started)? ________________

a.6) Please can you tell us about the nature of your “peer support” service/programme (for example, is it a parenting organisation, is it a service that has been developed by the neonatal staff/unit; is it an independent voluntary organisation, etc): **______________________________________**

a.7) How is your service funded? ____________________________________________

a.8) How many neonatal “peer supporters” work in your organisation/service in total? **_________**

a.9) Do the neonatal “peer supporters” get paid money for providing this service?

Yes – All / Yes – Some / No

a.10) Please could you tell us the professional backgrounds of all staff involved in the **management/coordination** (for example this would include all those who are on the board of directors or management committee) of your neonatal “peer support” service/programme (please click in all the correct boxes)?

| Clinical psychologist |  |
| --- | --- |
| Social worker |  |
| Experienced “peer supporter” |  |
| Parent |  |
| Neonatologist |  |
| Neonatal nurse |  |
| Midwife |  |
| Paediatrician |  |
| Other – please state |  |

a.11) Have the neonatal “peer supporters” in your service had their own experience of having a sick/premature child that was admitted/cared for in a neonatal unit (please click in the correct box)?

| Yes - all of them |  |
| --- | --- |
| Yes - some of them |  |
| No - none of them |  |

a.12) Do the neonatal “peer supporters” have a formal, written job description? Yes / No

**B. Identifying and Recruiting Neonatal “Peer Supporters”**

The following questions ask you about how neonatal “peer supporters” are identified and recruited into your “peer support” service/programme.

b.1) When recruiting neonatal “peer supporters”, does there have to have been a minimum period of time between the “peer supporter’s” own experience of having a sick/premature infant AND becoming a “peer supporter”? Yes / No

If yes, how long would you expect this time difference to be (for example six months, one years, two years)? ______________

b.2) How do you recruit neonatal “peer supporters” into your service (please click in all the correct boxes)?

| Advertise on the neonatal unit |  |
| --- | --- |
| Ask neonatal staff to identify ‘suitable’ parents |  |
| Advertise in local newspapers |  |
| Other – please describe |  |

b.3) Do you interview the neonatal “peer supporters”/parents before they are recruited into the service? Yes / No

If yes – who interviews the “peer supporters” (please click in all the correct boxes)?

| Neonatal healthcare professional |  |
| --- | --- |
| Counsellor |  |
| Psychologist |  |
| Social worker |  |
| Experienced “peer supporter” |  |
| Parent |  |
| Other – please state |  |

b.4) Do you explore if the neonatal “peer supporter” still has negative emotions about their own experience of having a premature/sick infant at recruitment? Yes / No

If yes, please can you describe how?

___________________________________________________________________________

___________________________________________________________________________

b. 5) Do the neonatal “peer supporters” have an opportunity to ‘shadow’ (to observe) experienced “peer supporters” **before** they provide support to parents? Yes / No

If yes, how long do they ‘shadow’ (observe) other “peer supporters” for? ________________

b.6) How do you assess if the neonatal “peer supporter” is appropriate/suitable to provide “peer support” (please click in all the correct boxes)?

| The parent/”peer supporter’s” performance (behaviour/responses) during the interview |  |
| --- | --- |
| The parent/”peer supporter’s” performance (behaviour/responses) during the training |  |
| Observation of the “peer supporter” providing support to parents |  |
| The “peer supporter” has to pass/show evidence of certain skills |  |
| Feedback from parents about the support they received from the “peer supporter” |  |
| Feedback from the “peer supporter” |  |
| Feedback from other “peer supporters” |  |
| Feedback from hospital staff |  |
| Other – please describe |  |

b.7) Have you had any situations when a neonatal “peer supporter” was not suitable/appropriate for this role? Yes / No

If yes – please could you tell us more about what happened/how you responded to this situation?

___________________________________________________________________________

___________________________________________________________________________

**C. Providing Support to Parents**

c.1) Please could you answer the following questions to tell us what and how neonatal “peer support” is provided to parents.

1. When is neonatal “peer support” provided to parents (please click in all the correct boxes)?

| In the antenatal period/before the parents have their baby |  |
| --- | --- |
| During the hospital stay |  |
| After the infant has been discharged |  |
| Other - please describe |  |

1. Who do the neonatal “peer supporters” provide support to (please click in all the correct boxes)?

| Parents of sick/premature infants |  |
| --- | --- |
| Grandparents |  |
| Other family members |  |
| Healthcare professionals |  |
| Siblings/other children in the family |  |
| Other – please describe |  |

1. How do the neonatal “peer supporters” provide support to parents (please click in all the correct boxes)?

| One-to-one face to face contact |  |
| --- | --- |
| Face to face contact in a group situation |  |
| Telephone/text contact with parents |  |
| Helpline service |  |
| Support provided by social media (such as FaceBook) |  |
| Written feedback (such as answering questions sent to a webpage) |  |
| Providing written leaflets/newsletters |  |
| Organised events (such as scrapbooking, hosting dinners) |  |
| Other - please describe |  |

1. Where do neonatal “peer supporters” provide support to parents? (please click in all the correct boxes)?

| In parents’ homes |  |
| --- | --- |
| In community centres/locations |  |
| In the neonatal unit |  |
| In the postnatal/postpartum ward |  |
| Not applicable as **all** support is provided over the telephone/Social media/by written communication |  |
| Other - please describe |  |

1. What types of support do the neonatal “peer supporters” provide (please click in all the correct boxes)?

| Emotional support (such as listening to parents’ concerns) |  |
| --- | --- |
| Social support (such as social visits, going to appointments with parents, etc) |  |
| Information (such as giving information about the benefits of breastfeeding, telling parents where they can access help and support) |  |
| Practical support (such as helping parents to hold/provide care to their infants, infant feeding) |  |
| Other - please describe |  |

c.2) Do you ‘match’ neonatal “peer supporters” and parents - for example, do you select “peer supporters” to work with certain parents based on similar characteristics/backgrounds?

Yes – always / Yes – sometimes / No

If yes – please can you select the criteria you use to match the parent and ”peer supporter” by clicking in all the correct boxes?

| Age |  |
| --- | --- |
| Language |  |
| Geographical location |  |
| Social background |  |
| Ethnic background |  |
| Babies with similar issues |  |
| Gestational age at birth |  |
| Multiple babies |  |
| Other – please describe |  |

c.3) Do you try and provide ‘continuity’ for the parents where possible, for example, is a neonatal “peer supporter” expected (where possible) to support the same parent over a period of time?

Yes / No

If no, why not? _____________________________________________________________

**D) Training, Supervision and Mentoring**

The following questions explore what training is provided to neonatal “peer supporters”, as well as any supervision and mentoring opportunities.

d.1) Do the neonatal “peer supporters” have to complete a training programme before they provide support to parents? Yes – All of them / Yes – some of them / No

d.2) How long is the training programme (for example 5 hours, 10 hours, 30 hours): __________

d.3) What skills/learning opportunities are included in the training programme (please click in all the correct boxes)?

| Basic communication and listening skills |  |
| --- | --- |
| How to show empathy, warmth and understanding |  |
| Understanding what the “peer support” role is and when there should be other professionals involved |  |
| Understanding the expected and normal responses of parents who have premature/sick infants |  |
| How to identify parents who may be at risk of more serious mental health issues |  |
| Understanding the natural stages of grief/mourning and loss |  |
| Knowledge of hospital and community services (such as where parents can get other/different types of support) |  |
| How to provide practical support to parents (such as holding babies, skin to skin care, infant feeding) – please detail |  |
| Other – please describe |  |

d.4) Who provides the neonatal “peer support” training (please click in all the correct boxes)?

| Clinical psychologist |  |
| --- | --- |
| Social worker |  |
| Experienced “peer supporter” |  |
| Parent |  |
| Neonatologist |  |
| Neonatal nurse |  |
| Midwife |  |
| Paediatrician |  |
| Other – please state |  |

d.5) Do the neonatal “peer supporters” have to attend additional training (such as a hospital training/induction programme) or undergo certain security checks before they provide support to parents? Yes / No

If yes, please tell us which training/checks are undertaken by clicking in all the correct boxes:

| Hospital training/induction programme |  |
| --- | --- |
| Criminal records checks |  |
| Privacy/confidentiality training |  |
| Other – please describe |  |

d.6) Do you provide any other additional training/learning opportunities for “peer supporters”?

Yes / No

If yes, how often are they provided? ____________________________

Are “peer supporters” able to decide/choose what additional training is provided? Yes / No

d.7) Do you think that the “peer supporters” receive enough training (please click in the correct box)?

| Yes – very much |  |
| --- | --- |
| Yes – to some extent |  |
| No – not at all |  |

d.8) What, if any, additional training is needed (please write in the box)?

___________________________________________________________________________

d.9) If the “peer supporters” provide support on the neonatal unit/hospitals – are they able to visit the unit/hospital before they start providing support to parents?

Yes / No / Not applicable

d.10) If you have any further comments/views about the training, what works, what doesn’t work, what else is needed, please write them here:

___________________________________________________________________________

___________________________________________________________________________

d.11) Are the neonatal “peer supporters” able to share their own experiences of having a premature/sick infant? Yes / No

If yes – please can you tell us how by clicking in all the correct boxes:

| During the training programme |  |
| --- | --- |
| During supervision |  |
| During sessions with a trained counsellor |  |
| During organised social events with other “peer supporters” |  |
| Other – please describe |  |

d.12) Do you think that the neonatal “peer supporters” should have more opportunities to talk about/discuss their own experiences? Yes / No / Don’t know

d.13) What, if any, additional opportunities for neonatal “peer supporters” to discuss their own experiences should be provided – please write in the box below: _____________________________

d.14) Do you provide regular/formal supervision (for example, opportunities for the “peer supporter” to discuss their role, share any concerns, highlight training needs, etc) for the neonatal “peer supporters”? Yes / No

If yes – how often is supervision provided (for example, monthly, every three months)? __________

Who provides the supervision (please click in all the correct boxes)?

| Clinical psychologist |  |
| --- | --- |
| Social worker |  |
| Experienced “peer supporter” |  |
| Neonatologist |  |
| Neonatal nurse |  |
| Midwife |  |
| Paediatrician |  |
| Other – please state |  |

How is supervision provided (please click in all the correct boxes)?

| One to one basis |  |
| --- | --- |
| Group basis |  |
| Other – please describe |  |

d.15) Do you provide a mentor system for the “peer supporters” (for example, do “peer supporters” have the name/contact details of a professional who they can contact on a day-to-day basis if they have any concerns)? Yes / No

If yes, what is the profession/background of the mentor(s) (please click in all the correct boxes)?

| Clinical psychologist |  |
| --- | --- |
| Social worker |  |
| Experienced “peer supporter” |  |
| Neonatologist |  |
| Neonatal nurse |  |
| Midwife |  |
| Paediatrician |  |
| Other – please state |  |

d.16) If a “peer supporter” was upset /distressed by observing/hearing about parent’s experiences, what support would be available to them (please click in all the correct boxes)?

| To go to counselling (provided by the “peer support” service/organisation) |  |
| --- | --- |
| To go to counselling (provided by a separate organisation/service) |  |
| To contact their supervisor/mentor immediately |  |
| To make an appointment to speak to their supervisor/mentor |  |
| To contact other “peer supporters” |  |
| To discuss at their next planned supervision session |  |
| Other – please describe |  |

d.17) Do you think that the “peer supporters” are provided with enough emotional support (please click in the correct box)?

| Yes – very much |  |
| --- | --- |
| Yes – to some extent |  |
| No – not at all |  |

d.18) What, if any, additional emotional support is needed for “peer supporters” (please write in the box below)?

__________________________________________________________________________

**E. What works and doesn’t work for “peer support” services?**

We are very interested to find out what works, and what doesn’t work for “peer support” services. Have you faced any difficulties in providing neonatal “peer support” in your area/country and/or what has helped to carry out “peer support” services? Please tell us about any thoughts or views you have on these issues.

_________________________________________________________________________________

__________________________________________________________________________________

__________________________________________________________________________________

__________________________________________________________________________________

**Many thanks for answering these questions. If you would be willing to take part in an audio-recorded interview to discuss your answers in more depth, please include your name and contact details (email) below. Please note that all interviews will have to be undertaken in English.**

**Name: _______________________________________________________________**

**Contact (email): _______________________________________________________**

**Thank you for taking part in the survey**

**Questionnaire for neonatal “peer supporters"**

**Many thanks for your support with this study**

Please could you read the following statements and then click in the box to indicate if you have:

Read and understood the information in the information sheet (v.2, 18/7/2016), and have had an opportunity to ask/receive answers to any questions.

Understood that any personal information provided (such as the name of your service/contact information) will not be shared with anyone outside of the research team.

Understood that the findings will be used in reports, journal publications and presentations, but you will not be identified.

Understood that it is up to you if you want to take part or not - you do not have to answer all of the questions - and if you have provided your personal contact details, you will be able to remove your data from the study up until June, 2017.

*Please note that ticking the box indicates your consent to take part in this study:*

*If you would be willing for us to keep and use your anonymised data for teaching; further research/evaluation; presentations and publications; sharing with other people doing similar studies please click in the box:*

**A. Background Information:**

a.1) What is the name of your “peer support” service/organisation? ________________

a.2) In which country is your “peer support” service? ____________________________

a.3) Have you had your own experience of having a sick/premature infant that was cared for in a neonatal unit? Yes / No

If yes, when was this experience (please can you tell us the month/year)? ______________

a.4) Are you paid to work as a “peer supporter” (do you receive money to provide this support)? Yes / No

a.5) What is your gender? Male/Female

a.6) Do you have a formal, written job description? Yes / No / Don’t know

a.7) When did you become a “peer supporter” (please can you tell us the month/year when you started work as a “peer supporter”)? _______________

**B. Becoming a Neonatal “Peer Supporter”**

b.1) When you started working as a neonatal “peer supporter” – how long had it been since your premature/sick baby had been born (for example, 6 months, 2 years, etc)?____________

b.2) Please could you answer the following questions to tell us what and how you provide support to others:

1. When do you provide support to parents (please click in all the correct boxes)?

| Before the parents have their baby |  |
| --- | --- |
| During the hospital stay |  |
| After the infant has been discharged |  |
| Other - please describe |  |

1. Who do you provide support to (please click in all the correct boxes)?

| Parents of sick/premature infants |  |
| --- | --- |
| Grandparents |  |
| Other family members |  |
| Healthcare professionals |  |
| Siblings/other children in the family |  |
| Other – please describe |  |

1. How do you provide support to parents (please click in all the correct boxes)?

| One-to-one face to face contact |  |
| --- | --- |
| Face to face contact in a group situation |  |
| Telephone/text contact with parents |  |
| Helpline service |  |
| Support provided by social media (such as FaceBook) |  |
| Written feedback (such as answering questions sent to a webpage) |  |
| Providing written leaflets/newsletters |  |
| Organised events (such as scrapbooking, hosting dinners) |  |
| Other - please describe |  |

1. Where is the support provided to parents (please click in all the correct boxes)?

| In parents’ homes |  |
| --- | --- |
| In community centres/locations |  |
| In the neonatal unit |  |
| In the postnatal/postpartum ward |  |
| Not applicable as **all** support is provided over the telephone/Social media/by written communication |  |
| Other - please describe |  |

b.3) Are you able to work with/support the same parent(s) over a period of time? Yes / No

b.4) What types of support do you provide to parents (please click in all the correct boxes)?

| Emotional support (such as listening to parents’ concerns) |  |
| --- | --- |
| Social support (such as social visits, going to appointments with parents, etc) |  |
| Information (such as giving information about the benefits of breastfeeding, where parents can get help and support) |  |
| Practical support (such as helping parents to hold/provide care to their infants, infant feeding, etc) |  |
| Other - please describe |  |

**C) Training, Supervision and Mentoring**

The following questions ask about the training you have received as well as any supervision and mentoring opportunities.

c.1) Did you attend a training programme when you first became a “peer supporter”? Yes / No

c.2) How long was the training programme (for example 5 hours, 10 hours, 30 hours): _________

c.3) What skills/learning opportunities were included in the training programme (please click in all the correct boxes)?

| Basic communication and listening skills |  |
| --- | --- |
| How to show empathy, warmth and understanding |  |
| Understanding what your role is and when there should be other professionals involved |  |
| Understanding the expected and normal responses of parents who have premature/sick infants |  |
| How to identify parents who may be at risk of more serious mental health issues |  |
| Understanding the natural stages of grief/mourning and loss |  |
| Knowledge of hospital and community services (where parents can get other/different types of support) |  |
| How to provide practical support to parents (such as holding babies, skin to skin care, infant feeding) – please describe |  |
| Other – please describe |  |

c.4) Who provided the training (please click in all the correct boxes)?

| Clinical psychologist |  |
| --- | --- |
| Social worker |  |
| Experienced “peer supporter” |  |
| Parent |  |
| Neonatologist |  |
| Neonatal nurse |  |
| Other – please describe |  |

c.5) Did you have to attend additional training (such as hospital training/induction) or have any security checks undertaken before you provided support to parents? Yes / No

If yes, which training/checks were undertaken (please click in all the correct boxes)?

| Hospital training/induction programme |  |
| --- | --- |
| Criminal records/activity check |  |
| Privacy/confidentiality training |  |
| Other – please describe |  |

c.6) Have you been provided with any other additional training/learning opportunities? Yes / No

If yes, how often are they provided (for example, weekly, monthly)? ___________________

Are you able to decide/choose what additional training is provided? Yes / No

c.7) If you provide support to parents in the neonatal unit/hospital, were you able to visit the unit/hospital before you started to provide support to parents? Yes / No / Not applicable

c.8) Do you think that you received enough training before you started to provide support to parents (please click in the correct box)?

| Yes – very much |  |
| --- | --- |
| Yes – to some extent |  |
| No – not at all |  |

c.9) What, if any, additional training should be provided (please write in the box below)? ______

c.10) If you have any further comments/views about the training - such as the length, who provides it and what is covered in the training - please write them here:

___________________________________________________________________________

___________________________________________________________________________

c.11) Has your “peer support” service/programme provided you with any opportunities to discuss your own experiences of having a sick/premature infant? Yes / No

If no, do you wish that you had been able to talk about/discuss your experiences? Yes / No

If yes – when have you been able to talk about/share your experiences (please click in all the correct boxes)?

| During the training programme |  |
| --- | --- |
| During supervision |  |
| During sessions with a trained counsellor |  |
| During social events with other “peer supporters” |  |
| Other - please describe |  |

Have you found it helpful to talk/about share your experiences (please click in the correct box)?

| Yes – very helpful |  |
| --- | --- |
| Yes – helpful |  |
| Neither helpful nor unhelpful |  |
| No – not very helpful |  |
| No – not at all helpful |  |

What, if any, further opportunities for “peer supporters” to talk about/discuss their own experiences should be provided (please write in the box below)?

___________________________________________________________________________

___________________________________________________________________________

c.12) Do you have regular/formal supervision sessions (for example, to discuss your role, to discuss any concerns, highlight training needs, etc)? Yes / No

If no, do you wish that supervision was provided? Yes / No

If yes - who provides the supervision (please click in all the correct boxes)?

| Clinical psychologist |  |
| --- | --- |
| Social worker |  |
| Experienced “peer supporter” |  |
| Neonatologist |  |
| Neonatal nurse |  |
| Midwife |  |
| Paediatrician |  |
| Other – please state |  |

c.13) How often do you receive supervision (for example, monthly, every three months)? _______

c.14) How is supervision provided (please click in all the correct boxes)?

| One to one basis |  |
| --- | --- |
| Group basis |  |
| Other – please describe |  |

c.15) Overall, how useful are the supervision sessions (please click in the correct box)?

| Yes – very useful |  |
| --- | --- |
| Yes – useful |  |
| No – not very useful |  |
| No – not at all useful |  |

c.16) Do you think that “peer supporters” receive enough supervision? Yes / No

If you have any further comments about opportunities for/experiences of supervision, please write them here:

___________________________________________________________________________

___________________________________________________________________________

c.17) Do you have a ‘mentor’ – a named person/professional who you can contact on a day-to-day basis if you have any concerns or issues? Yes / No

If no – do you think that a mentor should be provided? Yes / No

c.18) What is the professional background of your mentor (please click in the correct box)?

| Clinical psychologist |  |
| --- | --- |
| Social worker |  |
| Experienced “peer supporter” |  |
| Neonatologist |  |
| Neonatal nurse |  |
| Midwife |  |
| Paediatrician |  |
| Other – please state |  |

c.19) How useful is the support from your mentor (please click in the correct box)?

| Yes – very useful |  |
| --- | --- |
| Yes – useful |  |
| No – not very useful |  |
| No – not at all useful |  |

If you have any further comments about opportunities/experiences of receiving support from a mentor, please write them here:

___________________________________________________________________________

___________________________________________________________________________

c.20) Are any social events organised for you and other “peer supporters” to meet each other and discuss your experiences of providing “peer support”? Yes / No

If no – do you wish that these were provided? Yes / No

If yes - how useful are these events (please click in the correct box)?

| Yes – very useful |  |
| --- | --- |
| Yes – useful |  |
| No – not very useful |  |
| No – not at all useful |  |

c.21) If you were upset/distressed by observing/hearing about parent’s experiences – what support would be offered/available to you from your “peer support” organisation (please click in all the correct boxes)?

| To go to counselling (provided by the “peer support” service/programme) |  |
| --- | --- |
| To go to counselling (provided by a separate organisation/service) |  |
| To speak to your supervisor/mentor immediately |  |
| To make an appointment to speak to your supervisor/mentor |  |
| To talk to other “peer supporters” |  |
| Be able to discuss any issues at your next planned supervision session |  |
| Other – please describe |  |

c.22) Do you think that “peer supporters” are provided with enough emotional support (please click in the correct box)?

| Yes – very much |  |
| --- | --- |
| Yes – to some extent |  |
| No – not at all |  |

What, if any, additional emotional support is needed (please write in the box below)? _____

If you have any further comments about the emotional support provided to “peer supporters” - please write them here:

___________________________________________________________________________

**E. What works and doesn’t work for “peer support” services?**

We are very interested to find out what works, and what doesn’t work for “peer support” services. Have you faced any difficulties in trying to provide “peer support” and/or what has helped you to provide to support to others? Please tell us about any thoughts or views you have on these issues

___________________________________________________________________________

___________________________________________________________________________

___________________________________________________________________________

**Many thanks for answering these questions. If you would be willing to take part in an audio-recorded interview to discuss your answers in more depth, please include your name and contact details (email) below. Please note that all interviews will have to be undertaken in English.**

**Name: _______________________________________________________________**

**Contact (email): _______________________________________________________**

**Thank you for taking part in the survey**
