# Supplementary material for: International insights into peer support in a neonatal context: A mixed-methods study
Source: PLoS One. 2019 Jul 31;14(7):e0219743. doi: 10.1371/journal.pone.0219743 (PMC6668779; doi:10.1371/journal.pone.0219743)
Supplement: S2 Appendix — (DOCX) [file pone.0219743.s002.docx]

| **S2 - Appendix: Survey responses from MCTs into the background, funding and peer supporters’ recruitment criteria and processes (n=26)** | | |
| --- | --- | --- |
|  | N | Percentage |
| ***How long has your peer support service/programme been in operation?*** | | |
| 0-5 years | 10 | 38.5 |
| 6-10 years | 11 | 42.3 |
| 11-20 years | 3 | 11.5 |
| 20+ | 2 | 7.7 |
| ***Do peer supporters get paid for providing this service?*** | | |
| Mixture of voluntary/unpaid and paid | 8 | 30.8 |
| All supporters are voluntary/unpaid | 18 | 69.2 |
| ***Have the peer supporters had experience of having a child in a neonatal unit?*** | | |
| All | 15 | 57.7 |
| Some | 11 | 42.3 |
| ***Is there a minimum period of time between the peer supporter’s own experience***  ***and becoming a peer supporter?*** | | |
| Yes | 14 | 53.8 |
| No | 12 | 46.2 |
| ***How is the service funded?**** | | |
| Hospital/Health Funds | 8 | 30.8 |
| Grants/donations | 12 | 46.2 |
| Hospital/health funds & grants and donations | 3 | 11.5 |
| No specific funding | 2 | 7.7 |
| Not recorded | 1 | 3.8 |
| ***How do you recruit neonatal peer supporters into your service?**** | | |
| Advertise on neonatal unit | 7 | 26.9 |
| Ask staff to identify suitable parents | 11 | 42.3 |
| Formal recruitment methods | 11 | 42.3 |
| Self-selecting/parents approach the organisation | 12 | 46.2 |
| ***How do you assess whether the peer supporter is suitable to provide peer support?**** | | |
| Interview | 24 | 92.3 |
| Via training programme | 19 | 73.1 |
| Observations | 19 | 73.1 |
| Pass certain skills | 11 | 42.3 |
| ‘Shadowing’ experienced peer supporters | 18 | 69.2 |
| Feedback from peer supporter | 14 | 53.8 |
| Feedback from other peer supporters | 17 | 65.4 |
| Feedback from wider staff members | 17 | 65.4 |
| Feedback from parents | 17 | 65.4 |
| ***Do you ‘match’ neonatal peer supporters and parents?*** | | |
| Yes (always or sometimes) | 19 | 73.1 |
| No | 7 | 26.9 |

* Multiple options could be selected
