# Supplementary material for: International insights into peer support in a neonatal context: A mixed-methods study
Source: PLoS One. 2019 Jul 31;14(7):e0219743. doi: 10.1371/journal.pone.0219743 (PMC6668779; doi:10.1371/journal.pone.0219743)
Supplement: S3 Appendix — (DOCX) [file pone.0219743.s003.docx]

| **S3 – Appendix: Survey responses from MCTs and peer supporters into the types and timing of peer support and the training, supervision/mentoring and emotional support provided to peer supporters** | | |
| --- | --- | --- |
|  | N | Percentage |
| **Training** |  |  |
| ***Do peer supporters complete a training programme?*** |  |  |
| Yes | 39 | 81.3 |
| No | 9 | 18.7 |
| ***Content of training programme** (n=37)** |  |  |
| Basic communication skills | 27 | 72.9 |
| How to show empathy/understanding | 29 | 78.4 |
| Understanding peer support role | 37 | 100 |
| Normal responses | 32 | 86.5 |
| Identifying parents at risk of mental health issues | 24 | 64.9 |
| Grief and loss | 22 | 59.5 |
| Practical skills | 14 | 37.8 |
| Knowledge of other services/support | 28 | 75.7 |
| No information provided | 2 | 8.7 |
| ***Who provides the training?** (n=37)** |  |  |
| Experienced peer supporter/member of the organisation | 34 | 91 |
| Social workers | 12 | 32.4 |
| Clinicians (i.e. neonatal, infant feeding, midwifery | 14 | 37.8 |
| Counsellors/psychologists | 14 | 37.8 |
| Parents | 5 | 13.5 |
| ***Is additional security or hospital induction training offered?*** |  |  |
| Yes | 28 | 58.3 |
| No | 20 | 41.7 |
| **Mentoring** |  |  |
| ***Do you provide a mentor for the peer supporters?*** |  |  |
| Yes | 41 | 85.4 |
| No | 7 | 14.6 |
| ***What is the profession/background of the mentor(s)?**** |  |  |
| All mentors are experienced peer supporter/member of the organisation | 10 | 24.4 |
| Mentors included a range of clinical and related professionals as well as peer supporters | 31 | 75.6 |
| **Supervision** |  |  |
| ***Do you provide regular/formal supervision for the neonatal peer supporters?*** |  |  |
| Yes | 33 | 68.8 |
| No | 15 | 31.2 |
| ***How is supervision provided?**** |  |  |
| One to one | 27 | 81.8 |
| Group | 24 | 72.2 |
| Both | 19 | 57.7 |
| ***Who provides supervision?**** |  |  |
| Experienced peer supporter | 20 | 60.6 |
| Other professional: e.g. psychologist, social worker | 13 | 39.4 |
| **Types and timing of peer support provided to parents** |  |  |
| ***When do you provide support to parents?**** |  |  |
| Antenatal period | 25 | 52.1 |
| During hospital stay | 44 | 91.7 |
| Postnatal period | 38 | 79.2 |
| Across the whole perinatal period | 22 | 45.8 |
| ***Who do you provide support to****?** |  |  |
| Parents of premature infants | 48 | 100 |
| Other family members | 31 | 64.6 |
| Grandparents | 31 | 64.6 |
| Siblings | 23 | 47.9 |
| Other health professionals | 22 | 45.8 |
| Others | 2 | 4.2 |
| ***How do you provide support to parents?**** |  |  |
| One-to-one | 42 | 87.5 |
| In groups | 34 | 70.8 |
| Via social media | 37 | 77.1 |
| By telephone/text | 35 | 72.9 |
| Via helplines | 21 | 43.8 |
| Though written information (leaflets, emails) | 30 | 62.5 |
| Through events (such as hosting dinners, scrapbooking, events for world prematurity day) | 27 | 56.3 |
| ***Where is the support provided?**** |  |  |
| Online | 4 | 8.3 |
| Neonatal/hospital | 37 | 77.1 |
| Parents homes | 13 | 27.1 |
| Community locations | 27 | 56.3 |
| No response | 1 | 2.1 |
| ***What types of support do you provide?**** |  |  |
| Emotional support | 45 | 93.8 |
| Information | 47 | 97.9 |
| Social | 22 | 45.8 |
| Practical support | 26 | 54.2 |
| ***Do you provide ‘continuity’ for the parents?*** |  |  |
| Yes | 42 | 87.5 |
| No | 6 | 12.5 |
| **Opportunities/availability for emotional support** |  |  |
| ***Are peer supporters able to share their own experiences of having a premature/sick infant?*** |  |  |
| Yes | 45 | 93.8 |
| N | 3 | 6.2 |
| ***When are peer supporters able to share these experiences*?*** |  |  |
| Supervision sessions | 29 | 64.4 |
| During the training | 34 | 75.6 |
| With a counsellor | 32 | 71.1 |
| During social occasions/events with peer supporters | 17 | 37.8 |
| ***What type of emotional support is available?*** |  |  |
| Counselling (within the organisation) | 20 | 43.4 |
| Counselling (external to the organisation) | 12 | 26 |
| Speak to supervisor/mentor immediately | 39 | 84.7 |
| Make appointment to meet with supervisor/mentor | 20 | 43.3 |
| Talk to other peer supporters | 33 | 71.7 |
| Discuss at next planned supervision | 19 | 41.3 |

* Multiple options could be selected
